# Introduction to the Special Issue: Technological Applications in Mental Health and Mental Health Services Research

**DOI:** 10.1007/s10488-024-01392-0

**Published:** 2024-06-05

**Authors:** Julian A. Rubel, Wolfgang Lutz, Leonard Bickman

**Affiliations:** 1 Osnabrueck University, School of Human Sciences, Clinical Psychology and Psychotherapy of Adulthood, D-49076 Osnabrueck, Germany; 2 University of Trier, Department of Psychology, Clinical Psychology and Psychotherapy, D-54286 Trier, Germany; 3 Florida International University, FL Vanderbilt University Nashville, Miami, TN USA

Developments in speed and accuracy of computer technology enable a broad range of innovative applications in mental health and mental health services research. These technologies substantially extend the kind of data that can be collected (e.g., social media usage), the circumstances data can be collected (e.g., passively in everyday life), the scale at which this data can be collected (e.g., continuously every millisecond), as well as the way in which this big-data can be analyzed (e.g., with artificial intelligence algorithms). The ubiquitous presence of personal electronic devices like smartphones and smartwatches as well as their interconnectedness with other objects from our everyday lives via the internet provides a rich stream of data about most peoples’ real-life. This dynamic digital fingerprint can tell us a great deal about the mental health of an individual as well as changes herein over time. Moreover, technological developments provide the possibility to automize previously complex and time-consuming classification tasks. This provides researchers with much larger datasets and opportunities for complex modeling approaches that need large amounts of data.

The seven papers featured in this special section cover a broad spectrum of technological applications, and can broadly be categorized into three categories: video therapy, measurements in daily life, and automated coding of psychotherapy sessions.

In the first paper of this special issue Edelbluth and colleagues (2024) investigate the fundamental question of how different psychotherapy processes (therapeutic alliance, coping skills, and emotional involvement) change when switching from an in person face-to-face to a video format. Most of the studies that have dealt with this question are only qualitative in nature or had small sample sizes (e.g., Bechtold et al., 2023; Beck-Hiestermann et al., 2021; Giordano et al., 2022). In the present paper the authors report a study in which the intervention group (*n* = 227) shifted from face-to-face psychotherapy to video therapy during the COVID-19 pandemic, while the control group (*n* = 227) was treated face-to-face before the pandemic. A longitudinal piecewise multilevel model was calculated for each in-session process. While the therapeutic alliance and coping skills significantly increased after switching from face-to-face psychotherapy to video therapy in both groups, emotional involvement did not significantly increase. It is noteworthy that coping skills were rated higher in the control group than in the intervention group. The authors conclude that switching from face-to-face psychotherapy to video therapy had no negative impact on therapeutic alliance and emotional involvement but on coping skills.

The next four papers deal with data from ecologically momentary (EMA) designs in which patients are asked to provide data several times per day over a certain period of time. Not only the predominance of papers in this special issue but also in the mental health literature in general attests to the fact that EMA designs have already been established as an important tool to better understand patients’ problems. However, not much is known about patients’, therapists’, and supervisors’ views about the implementation of EMA in psychotherapeutic treatments. To close this gap, Meglio et al. (2024) conducted semi-structured interviews with eight patients, eleven therapists, and five supervisors from a research project implementing an EMA monitoring system. The participants provided positive feedback on the study’s informational resources and the implementation of EMA. They emphasized the necessity of customizing the EMA to meet patient needs. While patients viewed the EMA as beneficial to their daily routines, they mentioned challenges in completing assessments in the morning. Patients and therapists reported a neutral or positive impact of the use of the monitoring system on treatment.

One of the potentials of data collected in daily life is a more nuanced understanding of why people feel a certain way in a certain moment. The second paper in this category, authored by Langener et al., tries to test this tenet and aims to investigate how aspects of social context predict positive and negative affect. In a student sample of *N* = 11, they collected intensive within-person data from multiple passive and self-report sources over 28 days. They integrated data from digital phenotyping, experience sampling methods (ESM), and egocentric networks to calculate individual random forest machine-learning models. They only reached low prediction accuracy, even when combining different methods. The average coefficient of determination over all participants ranged from − 0.08 to 0.3, which demonstrates a large amount of variance across people. In general, combining different predictors improved the predictive accuracy of mood for most participants.

The next two papers in this category both present an interactive feedback tool which are supposed to provide graphical reports of intensive longitudinal data in order to enhance clinical utility of this information. In the first of these two papers, Stadel et al. (2023) focus on social context and its two components social relationships and social interactions. Social relationships were captured with personal social networks (PSN) and daily social interactions with ESM. They described the development of the prototype (Part 1) and two evaluation studies. Semi-structured interviews with 23 students (Part 2) and a clinical focus group discussion with five psychotherapy patients (Part 3) were conducted. In both studies, the prototype was rated as useful. The study’s feedback session, involving social context assessment via PSN and ESM, was evaluated as insightful by participating students. However, its added value beyond the assessments remains uncertain. Patients in focus groups suggested feedback could aid clinicians and patient-clinician collaboration, but noted the prototype requires additional explanation and cannot stand alone. Therefore, it serves as a starting point for future research and clinical application.

In the second of these two papers, Rimpler et al. (2024) introduced an interactive online tool called FRED (Feedback Reports on EMA Data). FRED includes descriptive statistics, time-series visualizations, and network analyses on selected EMA variables. In their study, they used data of 867 participants that were queried for 85 consecutive days resulting in up to 352 observations per participant. Participants completed an average of 204 (58%) surveys of 352 EMA time points. The feedback reports can be accessed by the Shiny app. By making the code and infrastructure of FRED available, the authors are aiming to facilitate future use of personalized data reports.

The last two papers of this special issue focus on the potential of technology for automated coding, an advantage that has been envisioned a couple of years ago (e.g., Imel et al., 2015) and which has been used in more and more applications recently (e.g., Eberhardt et al., 2024). The first of these two papers use video recordings of psychotherapy sessions to automatically recognize emotions. Using this intensive data per session, Atzil-Slonim et al. (2023) investigate the relationship between emotional flexibility and depressive symptoms at the client level (*n* = 58) and the session level (*n* = 283). Session-by-session, depressive symptoms were measured using self-reports and facial expressions using a computerized emotion recognition system. The authors report that higher emotional flexibility was associated with lower depressive symptoms at the treatment level and session level highlighting the importance of emotional flexibility as a trait-like and a state-like characteristic of depression.

In the last paper of this special issue Lalk et al. (2024) employed Natural Language Processing (NLP) to identify session topics and associate them with symptom severity (*r* = .45) and alliance ratings (*r* = .20). Their analysis was conducted on an archival dataset of 552 psychotherapy session transcripts and 124 patients. Leveraging topic modeling based on transformer language models, they found superior performance compared to traditional Latent Dirichlet Allocation based topic modeling. Using explainable AI, topics about health and negative experiences predicted higher symptom severity and topics about psychotherapy framework and income predicted lower alliance. The authors note that a simple bigram model showed vastly better performance though with reduced interpretability. They outline that the current approach is not viable to replace questionnaire data, but more sophisticated models that monitor different processes may provide helpful feedback to therapists about the session processes in the future.

Together, these papers represent a diverse and innovative collection of research that underscores the vital role of technology in advancing mental health services. By exploring various technological applications this special section contributes to a broader understanding of how digital tools can be harnessed to improve mental health outcomes, enhance research methodologies, and expand the accessibility of mental health services. Through these contributions, we witness the unfolding of a new chapter in mental health services research, marked by the transformative potential of technology.
